# Ethanol and Acetate Acting as Carbon/Energy Sources Negatively Affect Yeast Chronological Aging

**DOI:** 10.1155/2013/802870

**Published:** 2013-08-25

**Authors:** Ivan Orlandi, Rossella Ronzulli, Nadia Casatta, Marina Vai

**Affiliations:** ^1^SYSBIO Centre for Systems Biology Milano, Università di Milano-Bicocca, Piazza della Scienza 2, 20126 Milano, Italy; ^2^Dipartimento di Biotecnologie e Bioscienze, Università di Milano-Bicocca, Piazza della Scienza 2, 20126 Milano, Italy

## Abstract

In *Saccharomyces cerevisiae*, the chronological lifespan (CLS) is defined as the length of time that a population of nondividing cells can survive in stationary phase. In this phase, cells remain metabolically active, albeit at reduced levels, and responsive to environmental signals, thus simulating the postmitotic quiescent state of mammalian cells. Many studies on the main nutrient signaling pathways have uncovered the strong influence of growth conditions, including the composition of culture media, on CLS. In this context, two byproducts of yeast glucose fermentation, ethanol and acetic acid, have been proposed as extrinsic proaging factors. Here, we report that ethanol and acetic acid, at physiological levels released in the exhausted medium, both contribute to chronological aging. Moreover, this combined proaging effect is not due to a toxic environment created by their presence but is mainly mediated by the metabolic pathways required for their utilization as carbon/energy sources. In addition, measurements of key enzymatic activities of the glyoxylate cycle and gluconeogenesis, together with respiration assays performed in extreme calorie restriction, point to a long-term quiescent program favoured by glyoxylate/gluconeogenesis flux contrary to a proaging one based on the oxidative metabolism of ethanol/acetate via TCA and mitochondrial respiration.

## 1. Introduction

Human aging is associated with a host of time-dependent changes which are the clear manifestation of the progressive decline in cognitive and physical functions of an organism. Albeit extremely complex, aging has turned out to be influenced by mechanisms and nutrient/energy sensing signaling pathways that show strong evolutionary conservation. In this context, the single-celled yeast *Saccharomyces cerevisiae*, exploited as a model system, has provided valuable insight by making it possible to adopt experimental approaches that are not always feasible in higher eukaryotic systems. For example, the nutritional and metabolic status of yeast cells can be diversely coordinated by the simple choice of cultural conditions. Glucose is the preferred carbon and energy source, but in its absence other substrates such as glycerol, ethanol, acetate, or even fatty acids can be used [1]. Thus, the yeast life cycle can integrate metabolic characteristics that are typical for rapid growing cells, storage cells, or highly metabolizing cells depending on nutrient supply.

In the field of aging-related research, replicative and chronological lifespan models have been described which offer the opportunity to study the aging process of both proliferating and postmitotic quiescent mammalian cells, respectively [2–4]. The chronological lifespan (CLS) is defined as the length of time that a population of nondividing cells survives in stationary phase. Viability over time is measured as the ability to resume mitotic growth upon return to rich fresh medium [5]. In a standard CLS experiment, yeast cells are usually grown in synthetic defined media containing 2% glucose [6] where the metabolism is characterized by a high glycolytic flux, glucose fermentation, and a negligible aerobic respiration. Upon glucose depletion, the diauxic shift occurs which results in a shift from fermentation to respiration of the C2 compounds previously produced. This shift involves a massive reprogramming of gene expression including genes which encode enzymes involved in gluconeogenesis, the glyoxylate and TCA cycles. Moreover, overall growth rate is dramatically reduced. Finally, when nutrients are fully exhausted, cell division stops, and the yeast culture enters a quiescent stationary phase [7, 8]. In the stationary phase, yeast cells display a survival-based metabolism characterized by low metabolic rates and upregulation of stress resistance resulting from the integrated responses of different signaling pathways [9].

CLS can be increased by calorie restriction (CR) which, in yeast, is generally imposed by reducing the glucose concentration in the initial growth medium [10–12] or by transferring postdiauxic cells to water (extreme CR) [5]. Moreover, inhibition/reduction activity of two pathways which sense nutrient availability, namely, TORC1-Sch9 and Ras-PKA ones, also extends CLS [13–16]. These signaling pathways lead in part to common downstream targets which include the protein kinase Rim15 and the transcriptional factors Msn2/4 and Gis1 [17–19]. These factors, besides regulating directly or indirectly stress defence mechanisms, control the accumulation/utilization of intracellular and extracellular carbon sources [20–23]. In particular, Gis1 regulates the accumulation of acetate, a metabolite involved in chronological aging [24]. Interestingly, lack of the NAD^+^-dependent deacetylase Sir2, the founding member of Sirtuins, further extends the CLS of long-lived mutants such as *sch9*Δ, as well as the CLS in water indicating that the sole presence of Sir2 can serve as a “blocker” of extreme longevity extension [25]. In addition, *SIR2* inactivation induces stress resistance and affects positively the metabolism of extracellular carbon sources such as ethanol and acetate [25, 26]. These two by-products of glucose fermentation which are metabolised by yeast cells during the post-diauxic phase have been proposed as extrinsic factors promoting chronological aging [25, 27]. In fact, in some long-lived mutants, as well as in short-lived ones, an inverse correlation between the amount of extracellular ethanol or acidic acid and their CLS has been found [25, 26, 28–30]. In line with this, genetic or metabolic (CR) interventions which drive yeast metabolism away from acetic acid production increase CLS [27, 31]. Furthermore, although some connections have been established between nutrient-sensing pathways and the proaging effect of acetic acid involving superoxide anion accumulation which inhibits quiescence [32], the mechanisms by which this compound (and also ethanol) reduces the CLS are still controversial [33].

Here we present results showing that both ethanol and acetic acid contribute to chronological aging. In this context, these compounds are not simply extrinsic toxic factors, but it is their metabolic utilization as carbon/energy sources which results in proaging effects. In particular, in extreme CR, their oxidative metabolism increasing respiration impairs mitochondrial functionality and negatively affects long-term cell survival.

## 2. Materials and Methods

### 2.1. Yeast Strains and Growth Conditions

All yeast strains with null mutations were generated by PCR-based methods in a W303-1A background (*MAT*a *ade2-1 his3-11,15 leu2-3,112 trp1-1 ura3-1 can1-100*): *fps1*Δ (*fps1*Δ::*KlLEU2*), *sir2*Δ (*sir2*Δ::*URA3*) [34], *icl1*Δ (*icl1*Δ::*KlLEU2*), and *pck1*Δ (*pck1*Δ::*KlLEU2*) [26]. Yeast cells were grown in batches at 30°C in minimal medium (Difco Yeast Nitrogen Base without amino acids, 6.7 g/L), supplemented with 2% w/v glucose. Auxotrophies were compensated for with a fourfold excess of supplements [25]. All strains were inoculated at the same cellular density (culture volume no more than 20% of the flask volume). Growth was monitored by determining cell number using a Coulter Counter-Particle Count and Size Analyser, as described [35]. Doubling time (Td) was obtained by linear regression of the cell number increase over time on a semilogarithmic plot. For pH measurements, small aliquots of expired media were removed from the culture, and pH was determined using a pH meter.

### 2.2. Metabolite Measurements and Enzymatic Assays

At designated time-points, aliquots of the yeast cultures were centrifuged, and both pellets (washed twice) and supernatants were frozen at −20°C until used. Glucose, ethanol, and acetate concentrations in the growth medium were determined using enzymatic assays (K-HKGLU, K-ETOH, and K-ACET kits from Megazyme).

Immediately after preparation of cell-free extracts, Pck1 and Icl1 activities were determined as previously described [26]. Total protein concentration was estimated using the BCA Protein Assay Kit (Pierce).

Final values represent the average of three independent experiments. Differences in measurements were assessed by Student's *t*-test. The level of statistical significance was set at a *P* value of ≤ 0.05.

### 2.3. CLS Determination

Survival experiments in expired medium were performed on cells grown in minimal medium (with a fourfold excess of supplements) of 2% glucose as described by [25]. During growth, cell number and extracellular glucose, ethanol, and acetic acid were measured in order to define the growth profile (exponential phase, diauxic shift, post-diauxic phase, and stationary phase) of the culture. Cell survival was monitored by harvesting aliquots of cells starting 72 h (Day 3, first age-point) after the diauxic shift (Day 0). CLS was measured according to [25] by counting colony-forming units (CFUs) every 2-3 days. The number of CFUs on Day 3 was considered the initial survival (100%). Survival was also monitored in the presence of 50 mM pyrazole (Sigma) which was added in the expired medium at Day 1 after the diauxic shift.

For survival experiments in water, at Day 1 cells were harvested, washed with sterile distilled water, and resuspended in a volume of water equal to the initial culture volume. Every 48 h, cells were washed with water and resuspended in fresh water to remove nutrients released by dead cells [5]. The pH of the water was adjusted to 3.2 since it was the pH value measured in the expired medium or to 5.6. Survival experiments in water containing ethanol, acetic acid, or both were performed essentially as described [25, 26, 29]. Treatments are outlined in the text.

For CLS determination in media-swap experiments, cells were grown in minimal medium of 2% glucose (with a fourfold excess of supplements) and at Day 1 after the diauxic shift, harvested by centrifugation. Cell pellets were washed and then resuspended in the filtered original medium or equivalently conditioned one of the indicated strain. Resuspension in media collected at Day 1 was also performed in the presence of 50 mM pyrazole. Viability was measured as previously described.

### 2.4. Respiration Assays

The oxygen consumption of intact cells was measured at 30°C using a “Clark-type” oxygen electrode in a thermostatically controlled chamber (Oxygraph System, Hansatech Instruments, Norfolk, UK). For all respiration assays, 2 mL of cell suspension at a concentration of 5 × 10^6^/mL were quickly transferred from the flask to the oxygraph chamber, and routine respiration was recorded. Data were recorded at sampling intervals of 1 s (Oxygraph Plus software, Hansatech Instruments, Norfolk, UK). Respiratory rates were determined from the slope of a plot of O_2_ concentration against time, divided by the cellular concentration. All assays were conducted in biological triplicate.

Index of respiratory competence (IRC) was also measured according to [36] by plating identical samples on YEPD plates and on rich medium of 3% glycerol (YEPG) plates. IRC was calculated as colonies on YEPG divided by colonies on YEPD times 100%.

## 3. Results and Discussion

### 3.1. Lack of Fps1 Channel Increases CLS

**Figure 1:**
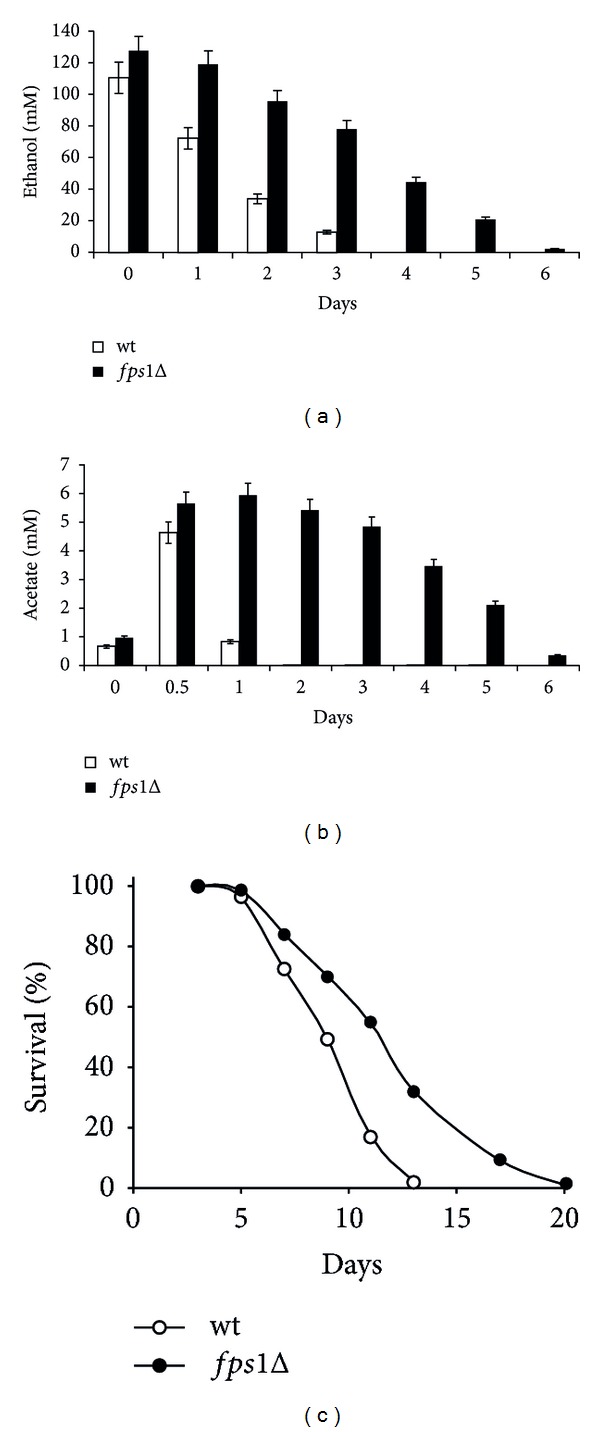
*FPS1* inactivation increases CLS in concert with a decreased uptake of ethanol and acetate. Bar charts of extracellular ethanol (a) and acetate (b) concentrations measured at the indicated time-points in wild type (wt) and *fps1*Δ mutant cultures during chronological aging. Day 0, diauxic shift. Data were obtained from mean values determined in three independent experiments. Standard deviations are indicated. (c) CLS of wt and *fps1*Δ mutant cells. At every time-point, viability was determined by counting CFUs on YEPD plates. 72 h after the diauxic shift (Day 3) was considered the first age-point (see Section 2). One representative experiment is shown.

Ethanol and acetic acid are two normal by-products of glucose fermentation, transiently accumulated in the yeast culture medium, which restrict CLS [25, 27]. Moreover, given the low concentration reached by acetic acid in the medium of chronologically aging cells and its faster exhaustion compared to that of ethanol, its physiological relevance as an extracellular factor promoting chronological aging is a matter of debate [33]. In this context, as a first step, we examined the effects on CLS of abolishing the major route of entry into the cell of the undissociated acetic acid such as the Fps1 channel. Uptake of acetate is linked to an active transport for the dissociated form of the acid through the Jen1 and Ady2 transporters accompanied by passive/facilitated diffusion of the undissociated acid through the Fps1 aquaglyceroporin [37, 38]. The former is inducible and subjected to glucose repression [39, 40] while the passive transmembrane flux is strongly influenced by the pH of the medium. In fact, the acetic/acetate couple forms a buffer system in a dynamic equilibrium: at low pH the equilibrium increasingly favours the protonated form while at pH above the pKa of acetic acid (4.75) charged acetate anions prevail. As shown in Figures 1(a) and 1(b), measurements of extracellular ethanol and acetate revealed that, at the diauxic shift, in the *fps1*Δ culture the amount of these C2 compounds was slightly higher than that in the wild type (wt) culture, in line with exometabolome data obtained during glucose fermentation [41]. However, after the diauxic shift (respiratory metabolism) a significant effect was observed on the depletion of both ethanol and acetic acid which was reduced in the mutant. In particular, as opposed to the expected fast exhaustion of acetic acid in the wt medium (Figure 1(b) and [29]), in the *fps1*Δ mutant this compound decreased very slowly, and it was still present 6 days following the entry in the post-diauxic phase (Figure 1(b)), which is in agreement with the role for Fps1 in facilitating the diffusion of the undissociated acid. In fact, during this phase in which the pH of the medium dropped to values of 2.70 for the wt and 2.55 for the *fps1*Δ mutant at Day 6 (Table 1), acetic acid is substantially undissociated, and the diffusional entry into the cells is elevated. Upon *FPS1* deletion, mutant cells can only rely on the uptake of the low fraction of acetate anions by the active transporters. Interestingly, chronologically aging* fps1*Δ cells lived longer than wt (Figure 1(c)) despite a prolonged exposure to acetic acid and ethanol.

### 3.2. Inhibition of Ethanol Metabolism Increases CLS

**Figure 2:**
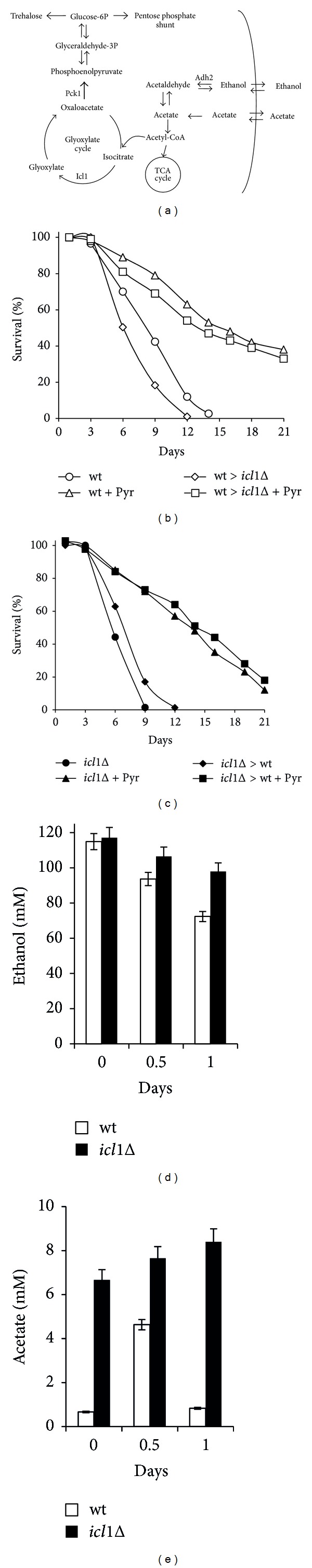
Pyrazole prevents the CLS shortening effect of ethanol. (a) Scheme of metabolic pathways allowing ethanol and acetate utilization. Only relevant reactions are shown. Adh2: alcohol dehydrogenase 2, Icl1: isocitrate lyase 1, Pck1: phosphoenolpyruvate carboxykinase 1. At Day 1 after the diauxic shift, pyrazole (50 mM) was added to the expired media of wt (b) and *icl1Δ* mutant (c) cells. In parallel, aliquots of cells were harvested and subjected to cell-free media swap with or without pyrazole. At every time-point, viability was measured as in Figure 1(c). One representative experiment is shown. Extracellular ethanol (d) and acetate (e) concentrations determined in the wt and* icl1Δ* cultures at Day 1. Day 0, diauxic shift. Standard deviations are indicated.

During chronological aging, after the diauxic shift, ethanol which is the main by-product of glucose fermentation, is metabolised by virtually the same pathway as acetate. In fact, after its oxidation to acetaldehyde by alcohol dehydrogenase 2 (Adh2), it is converted to acetate. Subsequently, acetate is activated into acetyl-CoA which is used to fuel the glyoxylate and TCA cycles (Figure 2(a)) [42, 43]. Consequently, we wondered whether blocking the main pathway for acetate production might influence the chronological survival of wt cells in their exhausted medium. To this end, after the diauxic shift when cells began to utilize the excreted ethanol, pyrazole which is an irreversible inhibitor of Adh2 [44] was added to the culture medium and CLS monitored. As depicted in Figure 2(b), pyrazole treatment led to CLS extension. A similar salutary effect took place also when pyrazole was added to the culture medium of postdiauxic *icl1*Δ cells (Figure 2(c)). *ICL1* encodes isocitrate lyase (Icl1), which is one of the unique enzymes of the glyoxylate cycle. During growth on C2 compounds, this cycle plays an essential role for anaplerosis of oxaloacetate which is the substrate of the key gluconeogenic enzyme phosphoenolpyruvate carboxykinase (Pck1) (Figure 2(a)) [43]. In the context of a CLS standard experiment, *ICL1* deletion results in a short-lived phenotype and impairment in acetate utilization [26]. Furthermore, pyrazole treatments led to a very slight acidification in the expired media of the wt and *icl1*Δ cultures (Table 1) indicating that the extracellular acidic pH alone is not sufficient to chronologically age yeast cells. Since we had already observed that pyrazole was able to abrogate the shortening effect of ethanol on CLS extension following extreme CR such as incubation in water [26], this confirms that some aspects of ethanol metabolism and not its mere presence (it enters the cells by passive diffusion) negatively affect CLS. We next performed some media-swap experiments between wt and *icl1*Δ cultures. Both strains were grown in minimal medium, and, at Day 1 after the diauxic shift, cultures were centrifuged and media were exchanged. The *icl1*Δ preconditioned medium, which contained more ethanol and acetic acid compared with the wt preconditioned one (Figures 2(d) and 2(e)) shortened the CLS of wt cells (Figure 2(b)). This detrimental effect on wt viability was abolished upon pyrazole addition, and CLS increased to the same extent as that of chronologically aging wt cells in their original medium in the presence of pyrazole (Figure 2(b)). Moreover, the wt preconditioned medium extended the CLS of the short-lived *icl1*Δ mutant (Figure 2(c)). Inhibition of ethanol oxidation by pyrazole further extended the CLS of the mutant which resulted, also in this case, similar to that of the chronologically aging mutant in its original medium supplemented with the Adh2 inhibitor (Figure 2(c)). Together these findings may point to proaging signaling effects of the metabolic pathways involved in the utilization of ethanol/acetate as carbon and energy source(s) by chronologically aging cells. This is consistent with the proposed role for acetic acid as a physiological trigger of growth signaling pathways which by promoting entry into S phase in unfavorable conditions would lead, among other effects, to replication stress in chronologically aging cells [45]. A DNA replication stress would negatively affect CLS [32, 46], and in this context experimental manipulations inducing such a stress have been recently shown to determine the loss of the reproductive capacity of chronologically aging cells [47]. Moreover, replication stress promotes apoptosis [48, 49]: a highly regulated cellular “suicide” program which is also activated during chronological aging [50]. In addition, acetic acid represents a compound which triggers apoptosis in the presence of glucose [51–53] and in glucose-derepressed *ach1*Δ cells [29]. Ach1 is an enzyme involved in acetate metabolism, and its lack decreases CLS [29, 54]. Consequently, stimulation of growth induced by acetic acid after the diauxic shift in the lack of favorable conditions required for cell cycle progression would ultimately cause apoptosis.

### 3.3. Physiological Amount of Acetic Acid Reduces CLS

**Figure 3:**
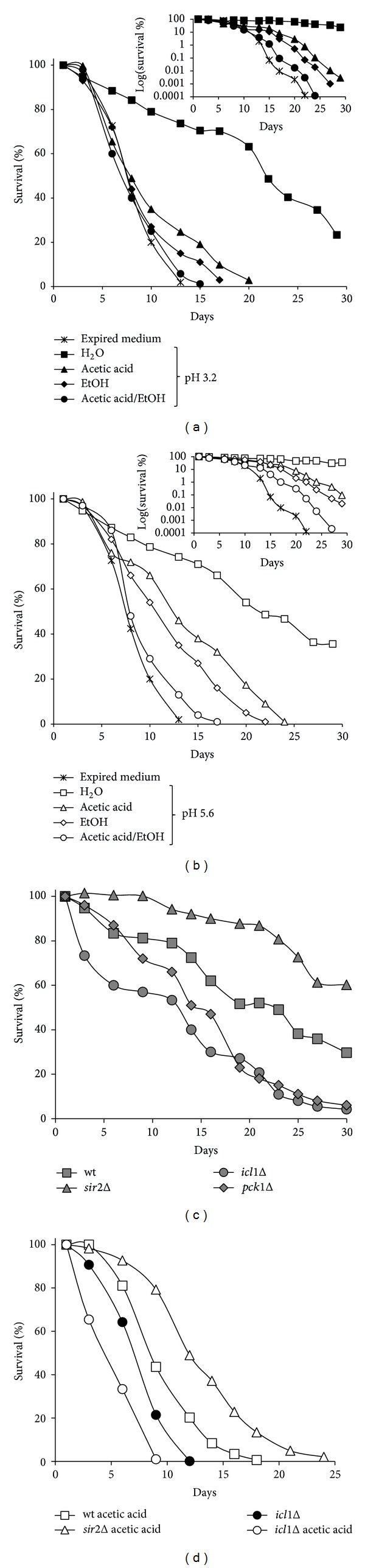
The glyoxylate-requiring gluconeogenesis plays a positive role in extreme life-span extension. At Day 1 after the diauxic shift, wt cells were switched to water adjusted to pH 3.2 (a) and to pH 5.6 (b) and challenged with ethanol (6 g/L), acetic acid (5 mM), or both. Every 48 h, cultures were resuspended in fresh water, and each time ethanol and acetic acid were added when indicated. At every time-point, viability was measured. Survival of cells in their expired medium was also monitored as control. Insets: CLS plotted on a log scale. One representative experiment is shown. (c) CLS of wt, *icl1*Δ,* pck1*Δ, and *sir2*Δ cells switched to pH 3.2 water at Day 1 after the diauxic shift. (d) In parallel, the indicated cultures were challenged with 5 mM acetic acid as in (a). Survival of *icl1*Δ cells in their exhausted medium was also monitored. One representative experiment is shown.

Next, we evaluated whether the physiological amount of acetic acid accumulated as a by-product of glucose fermentation could influence the chronological survival of yeast cells associated with their transfer to water, which is the extreme condition of CR known to extend CLS [25]. Therefore, we monitored the CLS of wt cells that, after the diauxic shift, were switched from expired medium to water supplemented with the amount of acetic acid (5 mM) we had detected in the expired medium (Figure 1(b) and [26, 29]). Treatments were performed in water whose pH was adjusted to 3.2 (the pH of the expired medium we measured) and in water buffered to pH 5.6. In the former condition the uptake of acetate is facilitated compared with that at pH 5.6 where the amount of the acetate anion considerably increases. As shown in Figure 3(a), the addition of 5 mM acetic acid to low pH water reduced CLS, but to a lesser extent than that elicited by ethanol [25, 26] which also in this case was supplied in amount comparable with that found in the expired medium. It is noteworthy that the addition of these C2 compounds together prevented CLS extension associated with transfer to low pH water resulting in a CLS similar to that of chronologically aging cells in their exhausted medium (Figure 3(a)). This suggests that it is a combined proaging effect of both metabolites which influences the CLS. Buffering water to pH 5.6 did not result in a CLS substantially different from that observed at pH 3.2 while the negative effect on chronological survival linked to the presence of acetic acid, ethanol, or both these compounds together was reduced (Figure 3(b)). Thus, buffering the extracellular medium alone is not sufficient to induce the fully extension of CLS observed in water, in line with data showing that an acidic environment alone is not sufficient to suppress the CLS extension associated with a CR regimen of growth which reduces acetic acid production [27, 29]. This further confirms that acidification accelerates chronological aging by influencing acetic/acetate equilibrium and consequently acetate uptake.

In the chronological aging paradigm, a proaging role is played by Sir2 which has as nonchromatin substrate the Pck1 enzyme. *SIR2* inactivation increases acetylated Pck1 in concert with increased enzymatic activity [26, 28]. Since this correlates with an enhanced glyoxylate/gluconeogenic flux and with a more efficient acetate utilization [26], we analyzed whether the addition of 5 mM acetic acid could influence the CLS of *sir2*Δ cells that, after the diauxic shift, were incubated in low pH water. In parallel, the same analysis was performed for the *icl1*Δ mutant. As shown in Figure 3(c), the effect produced by the single *SIR*2 and *ICL1* deletions on the CLS in water was the opposite. In fact, lack of Sir2 significantly extended the CLS compared with that of wt cells in agreement with [25–28] while lack of Icl1 reduced it. Interestingly, a similar decrease in cell survival has been observed following *PCK1* deletion ([28] and Figure 3(c)). Moreover, acetic acid-back *sir2*Δ cultures lived longer than acetic acid-back wt ones (Figure 3(d)). On the contrary, chronological survival of *icl1*Δ cells was affected dramatically by the same amount of acetic acid (Figure 3(d)), indicating that acetic acid, at this concentration, becomes extremely toxic for cells with an impaired glyoxylate cycle activity. Taken together these data suggest that the glyoxylate-requiring gluconeogenesis and the cell ability to metabolize acetate play positive roles in the CLS extension linked to extreme CR.

### 3.4. Ethanol Reduces Glyoxylate/Gluconeogenesis and Enhances Respiration of Cells in Extreme CR

**Figure 4:**
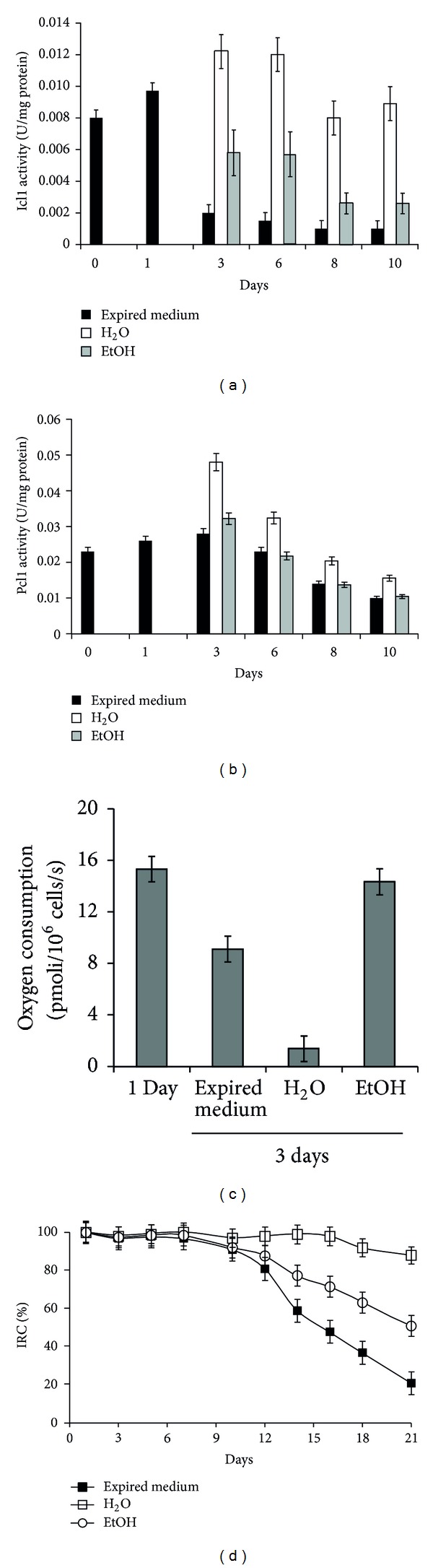
Ethanol affects the glyoxylate-requiring gluconeogenesis and the respiration of cells in extreme CR. At the indicated time-points, Icl1 (a) and Pck1 (b) enzymatic activities were measured in wt cells during chronological aging in their expired medium and after the switch to water or water/ethanol as in Figure 3. Day 0, diauxic shift. (c) Cellular respiration of the same cells in the indicated experimental conditions. Error bars are the standard deviation of three replicates. (d) Chronologically aging wt cultures at the indicated time-points were serially diluted, plated onto YEPD and YEPG plates, and the index of respiratory competence (IRC) was determined. Standard deviations of three independent experiments are indicated.

Starting from the aforementioned results, for the purpose of investigating the connection between the glyoxylate-requiring gluconeogenesis and chronological longevity we measured the enzymatic activity of Pck1 and Icl1 in chronologically aging wt cells in their expired medium or transferred to water. In parallel, we also examined cellular respiration. In fact, it is well known that in the former experimental condition, when glucose is depleted, cells consume the earlier produced ethanol/acetate via gluconeogenesis (Figures 4(a) and 4(b)), and concomitantly they increase their respiration (Figure 4(c)). In the extreme condition of CR, once cells were switched to water, the levels of Icl1 and Pck1 enzymatic activities increased and remained higher than those detected during aging in the expired medium (Figures 4(a) and 4(b)). In addition, they barely respired (Figure 4(c)). It is noteworthy that when these cells were challenged with ethanol, Icl1 and Pck1 enzymatic activities were reduced (Figures 4(a) and 4(b)), and the cellular respiration increased (Figure 4(c)). Similar results (with reduction and increase to a lesser extent) were obtained when acetate substituted for ethanol (data not shown) indicating that both C2 compounds are metabolised by the CR cells. Since the ability to respire relies on functional mitochondria and a direct correlation between reduced CLS and dysfunctional mitochondria has been reported [6, 55], we decided to analyze the index of respiratory competence (IRC). This index measures the percentage of viable cells which are competent to respire [36]. At Day 1, the IRC was about 100% for chronologically aging cells in the exhausted medium, in water, and in water/ethanol (Figure 4(d)) indicating that all the cells are respiration competent. Starting from Day 12, this value began to decrease progressively for the cells in the exhausted medium and for those in water/ethanol reaching about 20% and 50%, respectively by Day 21 which is indicative of a time-dependent loss of mitochondrial functionality. On the contrary, in the extreme CR condition the IRC was still about 80% (Figure 4(d)) indicating, on the one hand, that the low level of respiration is not due to impairment in mitochondrial functionality and, on the other hand, that resuspension in water exerts a protective role on mitochondria which become more prone to damage following ethanol addition.

To this effect, a causative role in inducing mitochondrial dysfunction is played by reactive oxygen species (ROS), and, at the same time, mitochondrial dysfunction leads to increased ROS formation [56]. Moreover, mitochondria are the major intracellular source of potentially harmful ROS such as the superoxide anion. This radical can directly induce oxidative damage or can be converted to other ROS which, in turn, induce aging-associated damage [57]. Chronological aging in the absence of any extracellular nutrient, namely, water, which correlates with an increased CLS, implies that cells have to establish a survival-based metabolism where energy is conserved by shutting down expensive growth-promoting pathways and concomitantly stress resistance and access to alternate energy stores are provided. In addition, cells have to limit damage to cellular components. In this context, reducing respiration may be beneficial since, although highly efficient in producing ATP, the oxidative metabolism produces the superoxide anion which is generated in the electron transport chain.

The other feature of cells in extreme CR discovered was an increase in the enzymatic activities of Pck1 (the main flux-controlling step of gluconeogenesis) and Icl1. This feature, combined with the fact that loss of their function blocks CLS extension, further supports the notion of a positive crucial role of glyoxylate/gluconeogenesis in the control of this form of longevity [28]. Increasing glyoxylate/gluconeogenesis may be advantageous to improve survivability during chronological aging in water since gluconeogenesis switches the direction of metabolite flow towards the biosynthetic precursor, glucose-6-phosphate, which is also needed for glucose stores (Figure 2(a)). In particular, trehalose has been proposed as the carbohydrate of choice for surviving starvation and upon cell cycle reentry from quiescence [58]. Moreover, hexoses generated from gluconeogenesis can be used via the pentose phosphate pathway generating additional NADPH which is essential for the activity of antioxidant defenses [59]. On the other hand, with regard to the glyoxylate pathway, it is important to recall that it does not only have the function of fueling gluconeogenesis but can contribute to NADH production [60].

This metabolic scenario may give some explanation why the CLS extension in water is intensified following *SIR2* inactivation [25]. In fact, the increase in the acetylated active form of Pck1 due to the lack of the Sir2-targeted deacetylation enhancing the glyoxylate/gluconeogenic flux [26] might further favour the establishment of a long-term quiescent program. On the contrary, the oxidative metabolism of ethanol/acetate via the TCA and mitochondrial electron transport chain increasing respiration may generate harmful ROS which impair mitochondrial functionality. This, in concert with induced growth signals in the lack of favorable conditions required for cell cycle progression [32], most likely negatively affects cell survival. Bearing in mind that the relationship between respiration, ROS, and CLS is very complex, how can the proaging effect induced by ethanol in nutrient starvation conditions fit with the ability of pregrowth on the same respiratory carbon/energy source to extend CLS [61, 62]? In fact, in addition to the role played by a mitochondrial respiratory threshold in regulating CLS [63], mitochondrial respiration affects chronological survival through ROS generation. They can be either deleterious or beneficial depending on the biological context/phases of the yeast cell cycle in which they are produced [57]. Although mitochondrial ROS have been associated with damaging effects which promote and/or accelerate chronological aging [64], they also function as signaling molecules with hormetic effects on longevity [65, 66]. In particular, elevating mitochondrial ROS during yeast exponential growth elicits an adaptive response which promotes CLS extension [67]. Similarly, the effects on CLS observed following growth on ethanol [61, 62] are also in line with an adaptive mitochondrial longevity signal generated during active growth which contributes to establishment of a better quiescent program.

## Figures and Tables

**Table 1:** pH values of exhausted media.

Days	wt	*fps1*Δ	*icl1*Δ	wt + pyrazole	*icl1*Δ + pyrazole
0	3.21 ± 0.07	3.20 ± 0.06	3.11 ± 0.06		
1	3.18 ± 0.04	3.16 ± 0.03	3.08 ± 0.05	3.18 ± 0.05	3.08 ± 0.07
2	3.13 ± 0.06	3.08 ± 0.04	2.98 ± 0.05	3.11 ± 0.04	2.89 ± 0.04
3	2.97 ± 0.05	2.90 ± 0.04	2.86 ± 0.06	2.93 ± 0.06	2.75 ± 0.07
4	2.81 ± 0.03	2.68 ± 0.06	2.68 ± 0.04	2.76 ± 0.07	2.63 ± 0.06
5	2.72 ± 0.06	2.56 ± 0.06	2.53 ± 0.06	2.65 ± 0.04	2.48 ± 0.07
6	2.70 ± 0.06	2.55 ± 0.05	2.49 ± 0.07	2.63 ± 0.07	2.43 ± 0.07

pH of the exhausted media was measured starting from diauxic shift, Day 0. Data presented are the mean values of three biological replicates. Standard deviations are indicated.
